# 青年肺癌左全肺术后肌肉及皮肤等全身多处转移1例

**DOI:** 10.3779/j.issn.1009-3419.2014.01.08

**Published:** 2014-01-20

**Authors:** 康宝 孔, 光虎 李, 成斌 张, 永生 崔

**Affiliations:** 1 130000 吉林，吉林大学第一医院胸外科 Department of Thoracic Surgery, the First Hospital of Jilin University, Jilin 130000, China; 2 130000 吉林，吉林大学第一医院病理科 Department of Pathology, the First Hospital of Jilin University, Jilin 130000, China

肺癌是目前全世界最常见的恶性肿瘤，其发病率及死亡率均居全球恶性肿瘤首位。近年来我国的肺癌发病率仍呈上升趋势。然而，低龄（< 30岁）肺癌患者在临床上仍较为少见。本文报道1例28岁低分化鳞癌患者行左全肺切除术，并且术后2个月内肾上腺、骨、肌肉、皮肤等多处转移，结合既往文献对青年肺癌进行回顾分析，更直观地反映出青年肺癌发展迅速，恶性度高、转移快的特点。

## 1 病历资料

患者，男，28岁，既往体健，无抽烟饮酒史，于半月前无明显诱因出现间断性咳嗽、咳痰，咳嗽阵发性发作，刺激性干咳为主，痰白色，泡沫样，量不多，易咳出，近5天出现喘息，夜间明显，偶有痰中带血，给予抗炎、止咳、解痉及化痰等治疗，近1天喘息症状加重遂住院。入院查肺部CT提示：胸段脊柱略右偏；双侧胸廓对称，纵隔气管略左移，右肺各叶段支气管开口通畅；左肺下叶支气管开口处管腔内及周围见团片状高密度影，并向外膨出，邻近舌叶支气管呈受压改变，病变边界不清，密度不均，最高CT值可达82 HU，远端支气管未见明确显示，所属肺组织体积缩小，密度增高，但相当于基底段肺组织略呈弧形外凸性改变，其内密度均匀；右肺各叶及左肺上叶、舌叶散在斑片状、条片状高密度影，边缘模糊；左下肺门影饱满；纵隔内未见明显肿大淋巴结影（图 1）。支气管镜检查提示：左主支气管末端见新生物生长，表面见毛细血管扩张，局部有白苔附着，质脆，触之易出血，管腔近闭塞（图 2）。于左主支气管末端行新生物活检，病理诊断：符合低分化鳞状细胞癌伴神经内分泌分化（图 3）。患者心电图、心脏彩超、腹部彩超、颅脑CT及骨扫描均未见异常，肺功能示：重度混合型通气功能障碍，FEV1实测值1.02，占预计值24%，小气道功能异常。患者状态差，不能很好配合，弥散功能测不出，肺储备率为52%。血气分析：PO2 79 mmHg，PCO2 45 mmHg，氧饱和度95%。患者诊断明确，根据全科会诊符合手术指证，于全麻下行左全肺切除及淋巴结清扫术，术中见肺门淋巴结肿大明显，上下肺静脉受侵，于是打开心包，于心包内结扎上下肺静脉，完整切除左全肺，并清扫全部淋巴结，手术较顺利。术后病理回报：非小细胞肺癌符合低分化鳞状细胞癌伴神经内分泌分化（三个灶，肿物1，体积4.5 cm×3 cm×2.2 cm；肿物2，体积2 cm×1.5 cm×1.0 cm；肿物3，体积4.8 cm×2 cm×1.9 cm），脉管内见癌栓，神经可见癌浸润，支气管壁可见癌浸润，脏层胸膜可见癌浸润，支气管切缘及血管切缘未见癌，支气管旁淋巴结可见癌转移（2/4），送检各组淋巴结可见癌转移（4/9），其中5组（0/4），7组（2/2）9组（1/1）10组（0/1）11组（1/1）。pTNM：T3N2M0，病理分期：Ⅲa期（IASLC, 2009）。免疫组化：CD56（散在+）、CK5/6（+）、CK7（+）、Ki-67（+60%）、CgA（-）、P63（+）、TTF-1（灶状+）。患者术后恢复良好，顺利出院。术后2个月化疗期间行肾上腺增强CT提示双侧肾上腺可见梭形异常强化影，右侧大小为2.2 cm×1.3 cm，左侧大小为1.3 cm×0.8 cm，边缘强化，考虑转移瘤；前额部有一逐渐增大圆形隆起包块，大小约3 cm×3 cm（图 4）。穿刺病理：真皮及皮下可见恶性肿瘤浸润，为转移性低分化鳞癌，免疫组化：Ki-67（+40%）、CK7（+）、TTF-1（-）、P63(+)、Syn（-）、CgA（-）、CK56（少+），AE1/AE3+。磁共振左大腿提示：左侧股骨中下部内前方股中间肌下方见结节状不均匀等T1长T2信号影，大小约1.5 cm×2.3 cm×3.2 cm，邻近股骨骨皮质表面欠光整，病变周围软组织及肌间隙内见条片状长T2信号影。所示右侧股骨上段、中下段骨髓腔内见斑片状长T1长T2信号影，提示股骨转移；左侧大腿穿刺病理回报：穿刺组织可见鳞状细胞癌浸润，提示左侧股中间肌转移；肺部CT提示：胸骨体上端可见线状骨质不连续及片状低密度影，周围可见软组织密度影，考虑胸骨转移。化疗两个疗程后家属放弃治疗，目前仍在随访中。

**1:**
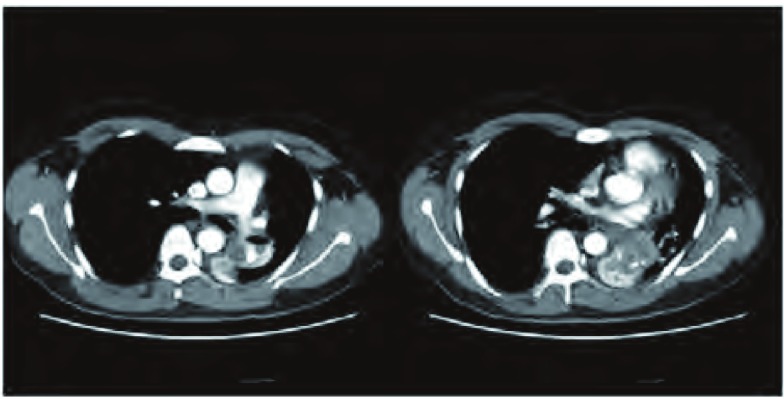
肺部CT：左肺下叶支气管开口处管腔内肿物及周围见团片状高密度影，伴左肺下叶肺不张。 Chest CT scan: A tumor is located in the left lower lobe bronchus openings and can see lamellar high-density shadow around it and atelectasis in the left lower lobe.

**2:**
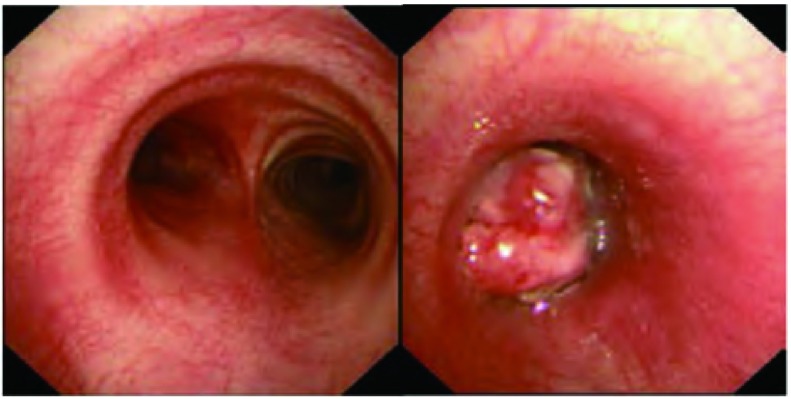
支气管镜示：左主支气管末端见新生物生长，管腔近闭塞。 Bronchoscopy showed neoplasm was in the distal end of left principal bronchus, and the cavity was almost occupyed completely.

**3:**
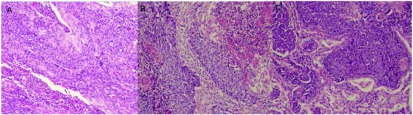
病理示：非小细胞肺癌符合低分化鳞状细胞癌伴神经内分泌分化（A为手术标本病理；B为前额部皮肤病理；C为左侧股中间肌病理）（HE，×200） Pathology demonstrate non-small cell lung cancer corresponding to poorly differentiated squamous cell carcinoma with neuroendocrine differentiation (A: Pathology of forehead skin; B: Pathology of surgical specimens; C: Pathology of left vastus intermedius.) (HE, ×200).

**4:**
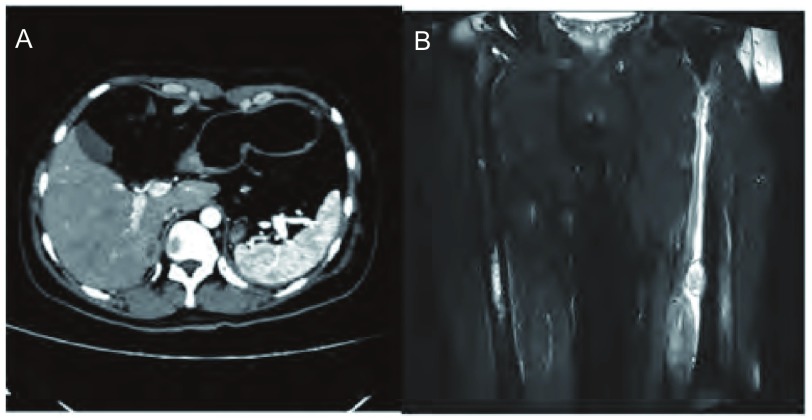
A：肾上腺增强CT提示：双侧肾上腺可见梭形异常强化影，右侧大小为2.2 cm×1.3 cm，左侧大小为1.3 cm×0.8 cm；B：磁共振左大腿提示：左侧股骨中下部内前方股中间肌下方见结节状不均匀等T1长T2信号影，大小约1.5 cm×2.3 cm×3.2 cm，所示右侧股骨上段、中下段骨髓腔内见斑片状长T1长T2信号影. A: Adrenal enhanced CT prompt fusiform enhancement in inside limbs of bilateral adrenal with size of 2.2 cm× 1.3 cm in the right and 1.3 cm×0.8 cm in the left; B: magnetic resonance of left thigh show nodosity uneven nodular T1 long T2 signal below vastus intermedius muscle in front of the inside part of the left femurl with size of 1.5 cm×2.3 cm×3.2 cm, and show pachy long T1 long T2 signal inside the upper and middle-lower bone marrow cavity.

## 2 讨论

近年来，肺癌的发生率逐年大幅度增加，据统计我国肺癌发生率已居于所有恶性肿瘤之首，且肺癌年轻化的趋势在明显增加，约占同期肺癌的1.0%-5.0%左右，国内外已有大量相关文献^[1-3]^报道，年龄多在30岁到40岁之间，但年龄小于30岁的更年轻肺癌病例则较少见文献报道^[4, 5]^。

本例患者以刺激性干咳为主要症状，偶有痰中带血，伴有渐进性呼吸困难，这与一般意义上中老年肺癌患者临床症状无明显差别^[6]^。但是本例患者从出现临床症状到确诊肺癌不足一月时间，这与大多文献报道相差较多。一方面可能是由于肿瘤生长速度快、倍增时间短，一般讲侵袭性高的小细胞肺癌倍增时间30天，鳞癌90天，大细胞肺癌120天，侵袭性高和侵袭性一般的腺癌为150天和180天以上^[7]^，但是肺癌倍增时间不仅与其组织学类型有关，还和其病理生长特点及分化程度有关，即分化差、实体生长倍增时间要短^[8]^，这些可能是青年人肺癌生长快、播散早、诊断时多为晚期、预后差的原因^[9-11]^。另一方面可能是肿瘤已长时间存在，但未引起症状，直至侵犯阻塞气管才表现出症状，以至拖延较长时间。故如何早发现、早诊断、早治疗成为青年肺癌患者避免延误治疗的关键。一旦出现刺激性咳嗽、痰中带血、胸痛等症状时，无论青年或者中老年都应及时行全面肺部检查。

另外，人们普遍认为吸烟与鳞癌的关系密切，即吸烟者中鳞癌的发生率高。本例患者无吸烟史，但值得注意的是，患者母亲于40多岁时死于肺癌，无其他家族肿瘤史。国内文献尚无有关肿瘤家族史与青年肺癌发病及预后相关报道，Lissowska等^[12]^报道青年肺鳞癌的发生与家族聚集性关系更为密切，这与我们大多认为早期肺腺癌与家族遗传有关矛盾，但该实验也未能证明家族遗传史在青年肺癌患者中扮演更重要角色。所以，与家族遗传是否有关有待进一步研究，且如能从分子生物学及遗传学等角度深入分析或许能对肺癌的诊断及治疗带来新的突破。

本例患者于全麻下行左全肺切除及淋巴结清扫术，术中见肺门淋巴结肿大明显，上下肺静脉受侵，只能打开心包，于心包内结扎上下肺静脉，并清扫全部淋巴结。术后病理回报：非小细胞肺癌符合低分化鳞状细胞癌伴神经内分泌分化。pTNM：T3N2M0，病理分期：Ⅲa期（IASLC, 2009），这也符合青年患者分化程度低、分期晚的特点^[11, 13, 14]^。青年肺癌手术切除率低，探查率高^[15, 16]^，其原因是：①无论是医生或是患者对肺癌警惕性小，以至拖延较长时间，发现时多为晚期；②青年人癌细胞生物活性强，常有外侵和转移，尤以腺癌和小细胞肺癌为主；③术前评估不足，术中见上腔静脉或心包受侵犯、广泛胸膜转移、肺门及纵隔淋巴结冻结无法分离。既往报道年轻肺癌患者病理类型以腺癌和小细胞肺癌居多，鳞癌相对少见^[4, 5, 9-11]^，在这些报道中，大多数患者已为中晚期肺癌，因为远处转移而失去手术机会。Tian等^[17]^报道可行手术治疗的青年肺癌中鳞癌占37%，全肺切除率达39%，5年生存率为46%，这些说明青年鳞癌患者如能及早发现，早期手术治疗，可能获得较好的预后。

有关青年肺癌患者的预后情况目前仍有分歧。相比同分期的老龄患者，青年患者对手术、化放疗具有更好的耐受性，采用更积极的联合治疗包括靶向治疗可能使之获得较好预后^[18]^。但从总体来看，由于就诊时多处于晚期，只有很少数患者可以得到根治性的治疗，大多数患者只能减轻症状，延长生存期，总体生存期与老年患者无明显差异^[1, 14]^。本例患者在术后短短2个月内相继出现肾上腺、前额皮肤、骨、肌肉等全身多处转移，充分证明了青年肺癌极高的恶性度，而且前额皮肤及左侧股中间肌转移瘤在临床上也极为罕见，查阅既往文献，尚无文献报道。

## 3 结论

通过该病例及查阅相关文献作者认为青年肺癌有以下特点：①发病率呈逐年增高趋势；②恶性度高，进展快；③误诊率高，手术率低；④预后差，生存率低。故作者有以下建议：①加强年轻人的健康体检，做到早发现、早治疗；②避免一切诱因，尤其是吸烟，不管是主动还是被动吸烟；③医务人员要加强对青年肺癌的认识，提高警惕；④从更深层次探求青年肺癌的发病机理，以求更综合的治疗，提高青年肺癌的远期生存率。
